# Complications After the Use of Trans‐Tibial Prostheses in Patients With Diabetes: A Cross‐Sectional Study

**DOI:** 10.1002/hsr2.72535

**Published:** 2026-05-18

**Authors:** Nasrin Moulodi, Sawza Saeed, Zekra Aziz, Mohammed Awla, Nabi Babakir, Rizgar Ahmad

**Affiliations:** ^1^ Prosthetics and Orthotics Department, Erbil Technical Health and Medical College Erbil Polytechnic University Erbil Iraq; ^2^ Rehabilitation Research Center, Department of Orthotics and Prosthetics, School of Rehabilitation Sciences Iran University of Medical Sciences Tehran Iran; ^3^ Erbil Physical Rehabilitation Center, International Committee of Red Cross Erbil Iraq; ^4^ Rania Center of Rehabilitation, Ministry of Health Sulaimania Iraq; ^5^ Duhok Specialized Center of Rheumatic Diseases and Medical Rehabilitation, Duhok General Health, Ministry of Health Duhok Iraq

**Keywords:** amputation, complications, diabetes mellitus, prostheses, satisfaction, vascular impairment

## Abstract

**Background and Aims:**

Many factors are affected after amputation and prosthetic use in patients with diabetes mellitus. Our study aimed to evaluate complications, satisfaction, and walking ability of patients with diabetes following 1 year of using trans‐tibial prostheses.

**Methods:**

The participants were diabetic patients with a history of using PTB‐SC transtibial prostheses design. Demographic and residual limb characteristics were collected. Twenty‐seven participants were included. A modified Orthotics Prosthetics Users Survey (OPUS) questionnaire, 2‐min walk test, and Visual Analogue Scale were used to evaluate satisfaction and complications, walking ability, and pain at rest, respectively. Descriptive statistics were used for analysis. Normality of the data was assessed using the Shapiro–Wilk test. The types of complications were grouped. The effect of the type of complication on walking ability and satisfaction was evaluated using one‐way analysis of variance. All statistical tests and calculations were performed using SPSS software.

**Results:**

The most common complications are put in four groups (group 1: volume change (11.1%), group 2: pain (during activity) (59.2%), group 3: redness(14.8%), and group 4: non‐complication(14.8%)). The mean for walking ability was 94.0 ± 36.7 m, satisfaction was 66.8 ± 5.8, and pain (at rest) was 20 (0,70). Pain (during activity) was the most common complication. The effect of type of complication on the walking ability and satisfaction, revealed no significant differences (*p* = 0.12, *p* = 0.057), with partial *ŋ* ^2^ = 0.21 and 0.27, indicating a large effect size, respectively. Post‐hoc comparisons using the Tukey HSD test indicated that the mean score for group 3 was significantly different from group 4 (MD: 11.1, SD: 3.9) in evaluating the effect of the type of complications on the satisfaction.

**Conclusion:**

This study provides valuable insights into the experiences of individuals with diabetic amputations. Addressing complications and optimizing prosthetic design, alignment, and fitting are essential for enhancing functional outcomes and improving the overall quality of life of this population.

**Abbreviations**
BMI: body mass index
ICRC: International Committee of the Red Cross
LLA: lower limb amputation
PAD: peripheral artery neuropathy
PRC: physical rehabilitation center
PTB‐SC: patellar tendon‐bearing supracondylar

## 1 Introduction

Diabetes mellitus is a global health issue affecting millions of individuals. This overall increase in prevalence contributes to an increase in the number of new cases of diabetes‐related complications, including peripheral artery neuropathy (PAD) and lower limb amputation (LLA) [1, 2, 3]. Amputations are frequently performed for various reasons. In cases where infection spreads from necrotic tissues, urgent amputation may be the only option to save a patient's life [4].

Diabetic complications are a major consequence of diabetes and account for a significant proportion of non‐traumatic LLAs worldwide [5]. Regaining mobility after complications is a critical rehabilitation goal. After LLA, only 48% of elderly amputees are fitted with a prosthesis. The ability to walk is contingent on the successful fitting of a prosthesis, which is partially influenced by comorbidities. Conversely, a patient's walking distance can affect the properties and components of the prescribed procedures [6, 7].

Individuals who have undergone amputation often find themselves less independent and reliant on others. This condition, in the long term, can lead to significant pain, restricted movement, and difficulties in performing daily tasks. In addition, a common distressing issue is phantom limb pain (PLP) and residual limb pain. Both are complex issues encompassing various concerns, from emotional well‐being to prosthesis fitting. Consequently, pain and individual experiences are significant factors influencing the overall amputation experience [8, 9].

Patient satisfaction is another vital indicator of the quality of care and plays a significant role in evaluating healthcare outcomes and managing healthcare budgets [10, 11, 12]. Satisfaction with the prosthesis influences the optimization of prosthetic use, reduces the likelihood of rejection, and enhances adherence to medical regimens [13, 14].

Trans‐tibial prostheses are often prescribed to restore mobility and improve quality of life. The successful use of these prostheses relies not only on effective prosthetic design and alignment but also on patient satisfaction and the management of potential complications [15]. In prosthetic management, for a better result, it is important to identify the primary complications experienced by diabetic patients. This research aimed to investigate the complications experienced by patients with diabetes using a patellar tendon‐bearing supracondylar (PTB‐SC) design and to adopt a multi‐faceted approach to provide recommendations for newly designed prostheses.

## 2 Methods

### 2.1 Study Location and Setting

This retrospective study assessed the key outcome measures and complications associated with the use of trans‐tibial prostheses in diabetic patients. This study was planned for patients who were attending the Erbil International Committee of the Red Cross (ICRC), Physical Rehabilitation Center (PRC), for rehabilitation. All of the electronic files that were registered in the center from January 2020 to December 2023, were checked and the file numbers of patients who had amputation were retrieved.

### 2.2 Participants and Sample Collection

The total number of all the amputations with different causes in the center was 352. From this population, the ones who faced trans‐tibial amputation by diabetes and had the opportunity to participate, because of transportation, were extracted. At the end, a total of 27 accepted the criteria and started to participate in the study. All subjects, who had the inclusion criteria, signed the informed written consent, and the study was approved by the Institutional review board of Medical Ethics Committee of Erbil Polytechnic University (24/0043 HRE). Trans‐tibial amputation secondary to diabetes, using prosthesis with PTB‐SC design for at least 1‐year and made from polypropylene material were the main inclusion criteria. Also, complications secondary to diabetes were considered, and the subjects didn't have complications that interfered with functional mobility, for example, blindness. This study conforms to all STROBE guidelines and reports the required information accordingly [16].

### 2.3 Data Collection

The primary outcome measures were complications, pain (at rest), walking ability, and satisfaction. A specific assessment form was designed for each participant to collect demographic data, activity level, residual limb shape and length (as secondary outcome measures) information, complications, and responses to the questionnaire. The Visual Analog Scale (VAS) was used to measure pain intensity at rest. This scale consists of a 100‐cm line with endpoints representing 0 (no pain) and 100 (the worst pain imaginable). Participants were asked to indicate their average pain over the past month by placing a mark on the line, resulting in a pain intensity score out of 100. In the second test, the participants were instructed to walk for 2 min, and the distance covered was measured. Two markers were placed on a 30‐meter‐long section of the floor, and participants were instructed to walk between these points. The examiner timed the walk and recorded the distance covered, noting the use of any assistive devices during the test [17, 18]. The third test involved filling out the modified Orthotics Prosthetics Users Survey (OPUS) questionnaire, along with a custom‐designed questionnaire to evaluate complications and satisfaction. OPUS is a reliable and valid self‐report instrument that facilitates the evaluation of patient outcomes. It can assess the functional status, quality of life and satisfaction with the orthotics and prosthetics. The modified version of the questionnaire that was used in this study consisted of 9 questions. Questions that were not relevant to this study were removed [19]. It has four answer options: strongly agree, agree, disagree, and strongly disagree. They are graded as 4–1, respectively [20]. The percentage of these grades is used for further analysis. At the time of study, there was no validated Kurdish version of the OPUS questionnaire available. Therefore, the items were interpreted and translated verbally by the assessor during the data collection process to ensure participants' understanding. Questions included statements such as “The weight of my prosthesis is manageable” and “My prosthesis is durable”. These inquiries facilitated the assessment of complications and overall satisfaction. The questionnaire and assessment form designed for this study are attached as Supporting Information S1.

### 2.4 Statistical Methods

All data were entered into IBM SPSS Statistics for Windows, Version 21.0 (IBM SPSS, Armonk, NY: IBM Corp). All statistical tests and calculations were performed using SPSS. Normality of data distribution was assessed using the Shapiro–Wilk test. For reporting demographic and secondary outcome measures, descriptive statistics were used [21]. In descriptive statistics, quantitative variables (demographic data and primary outcome measures) were analyzed by mean and standard deviation for normally distributed data and median for non‐normal distribution of data. Qualitative variables were presented as frequency and percentage. The characteristics of residual limb, as a qualitative variable, were classified into three sections: residual limb length (short, medium, and long), shape (conical, cylindrical, and bulbous); and activity level (low, moderate, and active). In addition, the type of complications was grouped. Also, the effect of the type of complication on walking ability and satisfaction was evaluated using one‐way analysis of variance (ANOVA). A *p* value ≤ 0.05 was considered statistically significant for all analyses.

## 3 Results

### 3.1 Demographic Characteristics of Study Participants

The total sample size of subjects who had trans‐tibial amputation because of diabetes and participated in the analysis was 27 (20 males and 7 females). No one was excluded from the study during the process. Fifteen amputations were on the right side and 12 on the left side. The mean age of participants was 61.1 ± 11.2 years. Overall, the body mass index (BMI) for these participants was 28.7 ± 4.9. Regarding residual limb characteristics, medium residual limb length, low activity level, and cylindrical shape of the residual limb are the most common types of amputation properties. The frequency and percentage of mentioned parameters are 18 (66.7%), 16 (59.3%), and 16 (59.3%) out of 27 (100%) subjects in each classification, respectively. Table 1 summarizes the characteristics of participants and residual limbs.

**Table 1:** Characteristics of participants and residual limbs.

Items	*N* of diabetic amputations	
	27	
Side (right/left)	15 right/12 left	
	**(Mean ± SD** a **)**	**Test of normality (*p* value)**
Age (years)	61.1 ± 11.2	0.4
Height (m)	1.7 ± 0.08	0.61
Weight (kg)	84.5 ± 18.3	0.16
BMI^b^ (kg/m^2^)	28.7 ± 4.9	0.94

Residual limb characteristics	Categories	Frequency (percent)
Stump length	Short	6 (22.2)
	Medium	18 (66.7)
	Long	3 (11.1)
Activity level	Low	16 (59.3)
	Moderate	11 (40.7)
	Active	0
Stump shape	Conical	1 (3.7)
	Cylindrical	16 (59.3)
	Bulbous	10 (37)

^a^ SD: standard deviation.

^b^ BMI: body mass index.

### 3.2 Primary and Secondary Outcome Measures Analysis

**Figure 1:**
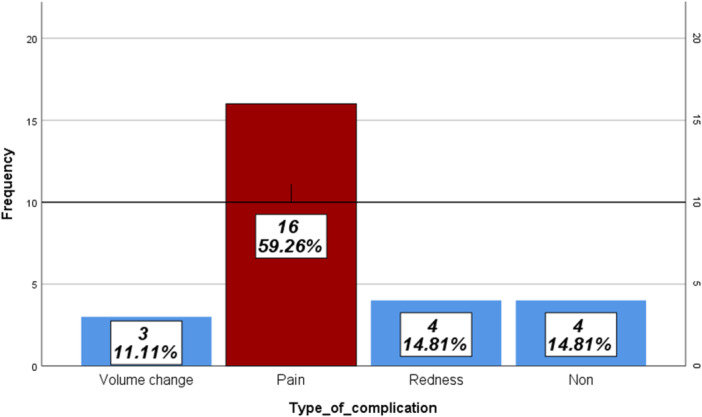
The description of frequency of different types of complications.

**Figure 2:**
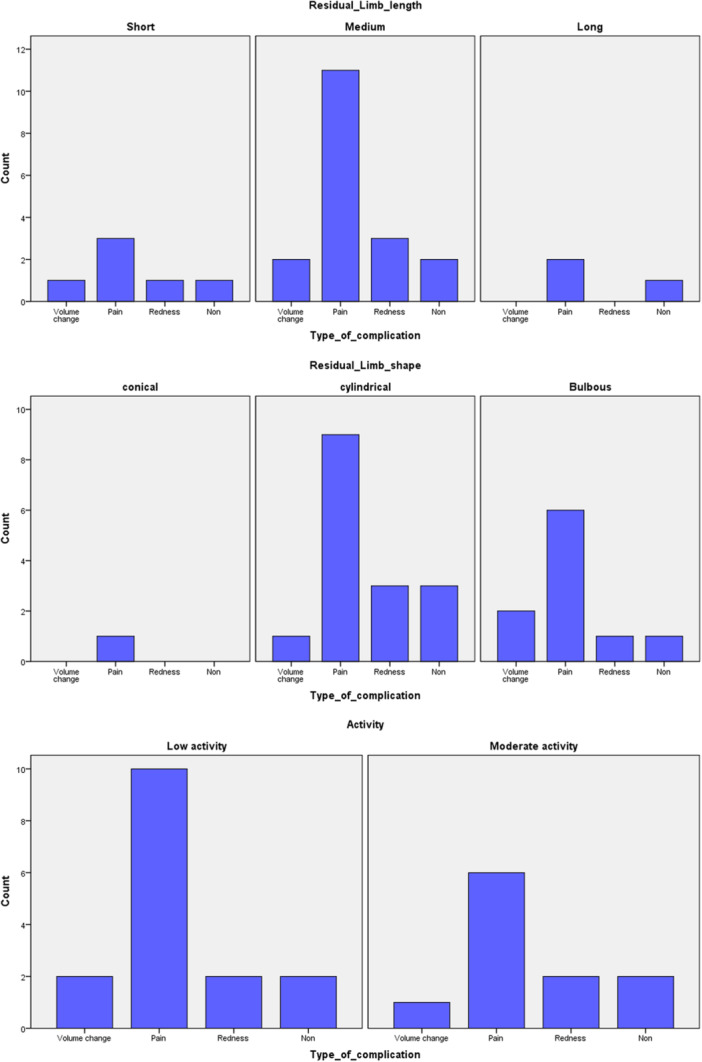
Distribution of residual limb characteristics (length, shape, and activity level) across different types of complications.

Pain at rest was 20 (0–70). The mean for walking ability was 94.0 ± 36.7. At the same time, the mean for the percentage of satisfaction was 66.8 ± 5.8. Based on the test of normality, pain at rest, showed non‐normal distribution (*p *< 0.001). After asking patients about their complications like volume change, pain during activity, redness, and non‐complications were the most frequent complications among participants (Figure 1). So, the complications were put in four groups (group 1: volume change, group 2: pain (during activity), group 3: redness, and group 4: non‐complication). There were no other types of complications in the studied participants. The distribution of residual limb characteristics across different groups is shown in Figure 2. Sixteen amputees (59.3%) experienced pain during activity. It is the most frequently reported complication in these subjects. Meanwhile, 4 amputees (14.8%) represented redness, and non‐complication, also showed in 4 amputees (14.8%). The remaining 3 amputees reported volume change during 1‐year of using prostheses. It accounts for 11.1% of participants (Table 2).

**Table 2:** The analysis of pain, walking ability, satisfaction, and description of complications.

Primary outcome measures		Test of normality (*p* value)
Pain (at rest) (median (amin − max))	20 (0–70)	c< 0.001
Walking ability (mean ± bSD)	94.0 ± 36.7	0.37
The percentage of satisfaction (mean ± SD)	66.8 ± 5.8	0.26

Type of complications	Frequency (percent)
Group 1: volume change	3 (11.1)
Group 2: pain (during activity)	16 (59.2)
Group 3: redness	4 (14.8)
Group 4: non‐complication	4 (14.8)
Total	27 (100)

^a^ minimum − maximum.

^b^ SD: standard deviation.

^c^ significant.

A one‐way between‐groups ANOVA was conducted to explore the impact of different groups of type of complication on the walking ability and satisfaction. The effect of the type of complication on the walking ability revealed no significant differences (*F*[3, 22] = 2.11, *p* = 0.12) between four groups of complications. The effect size, calculated, was partial *ŋ* ^2^ = 0.21. This amount indicates a large effect size. In addition, a trend toward a significant effect of the type of complications on patient satisfaction was seen (*F*[3, 22] = 2.89, *p* = 0.057). The effect size was large, with the type of complication accounting for 27% of variance in percent of satisfaction (partial *ŋ* ^2^ = 0.27). Post‐hoc comparisons using the Tukey HSD test indicated that the mean score for group 3 was significantly different from group 4 (MD: 11.1, SD: 3.9).

## 4 Discussion

This study aimed to evaluate the experiences of patients with diabetic amputation using a trans‐tibial prosthesis with PTB‐SC design. Data collection involved a comprehensive evaluation of demographic information, activity levels, residual limb characteristics, pain, walking ability, satisfaction, and complications. The demographic characteristics of the participants indicated a varied sample in terms of age, height, weight, and BMI. This diversity is crucial in understanding how prosthetic interventions may impact individuals across different age groups and body characteristics, and the results are more generalized [22]. Also, the characteristics of the residual limb show the effects and properties of the subjects, to be considered for the evaluation of results [23].

The current study found that pain (during activity), redness, and volume change were identified as the primary complications experienced by the participants. Pain was the most prevalent complication, affecting 59.3% of the subjects. These results are in line with those of Chatterjee et al., who reported discomfort in prosthetic users. Local stress concentration, friction and slippage, warm environment inside the socket and altered gait dynamics add to this discomfort. Skin irritation and redness occurred in 41% of trans‐tibial amputees [24]. In the other study, Hugberg et al. showed a close to half (51%) residual limb pain, phantom limb pain (48%), back pain (47%) and 46% in the other legs in the non‐vascular trans‐femoral amputees [25]. Nusrat Jahan also found secondary complications like a blister, skin irritation, erythema, residual limb volume change; and knee and hip pain in the long‐term lower limb prosthetic users [26]. But Courtney et al. emphasized on socket design, alignment and interface pressures as elements for maximize mobility and minimize injurious forces in the trans‐tibial prosthetic users [27]. Between pain and daily hours of prosthesis use, there is a significant correlation [28]. These findings highlight the need for ongoing monitoring and intervention to address discomfort and ensure optimal prosthetic usage [23]. All elements that have a role in increasing pain, as a main factor, should be investigated critically, and guidelines should be provided to the rehabilitation team [29].

Assessment of pain, walking ability, and satisfaction revealed important insights into the functional outcomes of the prosthesis. The median for pain (at rest) was 20 (0,70). This is less than half, and at a relatively low level of experiencing pain. It indicates that most participants experienced mild or no pain during rest. However, the wide range of pain scores suggests considerable inter‐individual variability, with a subset of patients reporting moderate to severe pain at rest. Because it is not related to the time that the prosthesis is used, and there are no forces from the prosthesis. However, pain and complications likely influence walking performance, as evidenced by the correlation analysis [9]. The mean of walking ability was 94.0 ± 36.7 per 2 min. This distance is without assistive devices and showed a considerable ability of participants using a prosthesis. Lower limb amputation not only affects people's ability to walk but may also impact their participation in valued activities, body image perception, and quality of life. Kwak et al. found that, after 3‐month gait training with prosthesis, short‐term walking outcomes were poor in diabetic compared to non‐diabetic amputees [30]. However, the percentage of satisfaction showed 66.8 ± 5.8. This amount of satisfaction shows a notable result in the prosthetic users of this study. It suggests a generally positive perception of the prosthetic intervention. Suraiya Akter, in a study on health and satisfaction level of patients with lower limb amputation, conclude 88% satisfaction with the prosthesis [31]. By considering the three important items of pain, walking ability and satisfaction, it is worth noting that the ability to walk will decrease due to increasing pain and complications. When the patient is not able to walk and perform activities independently, it will affect their satisfaction [32]. Satisfaction with prosthetic use is multifaceted and is influenced by various factors, including comfort, the existence of complications, and functionality [33].

The majority of participants had medium residual limb length and cylindrical shape, which are common characteristics of trans‐tibial amputations. In addition, low activity levels shape the other characteristics. Understanding residual limb morphology helps optimize prosthetic fit and comfort [34]. Davie‐Smith et al. concluded that diabetic amputations were younger and had a higher proportion of trans‐tibial amputation. This age is in contrast to the mean age in this study (mean ± SD = 61.1 ± 11.2). Also, successful limb fit was approximately similar in diabetic and non‐diabetic amputees [35]. These results reinforce the significance of demographic factors for decision‐making on prosthetic management. The association between different types of complications on one side and primary and secondary outcomes on the other side makes these associations clear. Although the effect of the type of complication on walking ability did not reach statistical significance, the observed effect size was large (partial *ŋ* ^2^ = 0.21), indicating a substantial practical impact. This discrepancy is likely attributable to the limited sample size and reduced statistical power, particularly given the division of participants into multiple complication subgroups. It is similar for the association between type of complications and satisfaction (*p* = 0.057, partial *ŋ* ^2^ = 0.21). As mentioned, the borderline statistical significance and large effect size in the effect of complications on the satisfaction, seemed to indicate that satisfaction may be more strongly influenced by the type of complication than walking ability.

In investigating the distribution of types of complications and residual limb characteristics, pain (during activity) was the most common item in the different categories of low activity, cylindrical shape, and medium length. It can be related to the finding that these three sections had the largest frequency among the others. It can have an effect, but the influence and the main cause of pain should be investigated. As found, redness and volume change have been seen. It has an impact on the proper fit of the socket, alignment, restriction of movement, and will increase the pain, accordingly.

The limitations of the study include a lack of application of intervention to decrease complications and conducting longitudinal follow‐up. Future research could benefit from these results by using trial and longitudinal designs to realize the long‐term effects of prosthetic interventions in diabetic amputations. Also, due to the small sample size, the findings of this study should be interpreted with caution and are not readily generalized to the wider population. The other limitation of the study was that the OPUS questionnaire was not available in a validated Kurdish version. The items were verbally translated and explained by the assessor during administration, which may have introduced interpretation bias and affected the consistency of responses. Our recommendation for the upcoming research is to consider these complications in the new cast and final prosthesis and analyze the results between the new prosthesis and the old one. However, the importance of follow‐up should be explained to the participants to reduce the complication rate periodically. Also, it is worth noting that recording the number of patients who chose not to undergo prosthetic fitting will be valuable for rehabilitation management of this population.

## 5 Conclusion

In conclusion, this study provides valuable insights into the experiences of individuals with diabetic amputations who use trans‐tibial prostheses. Despite experiencing complications such as pain, volume change, and redness, the participants demonstrated satisfactory walking ability and overall satisfaction with the prosthesis. Addressing complications and optimizing prosthetic design and fitting are essential for enhancing functional outcomes and improving the overall quality of life. Future research and clinical practice should focus on implementing comprehensive assessment protocols, managing complications effectively, and providing ongoing support to individuals using prostheses.

## Author Contributions

**Nasrin Moulodi:** conceptualization, methodology, software, supervision, formal analysis, investigation, visualization, project administration, resources, writing – original draft, writing – review and editing. **Sawza Saeed:** software, data curation, writing – review and editing, methodology. **Zekra Aziz:** methodology, writing – review and editing. **Mohammed Awla:** writing – review and editing, methodology, formal analysis, data curation, visualization. **Nabi Babakir:** methodology, data curation, writing – review and editing, resources, investigation. **Rizgar Ahmad:** data curation, methodology, writing – review and editing.

## Funding

The authors have nothing to report.

## Conflicts of Interest

The authors declare no conflicts of interest.

## Transparency Statement

The corresponding author, Nasrin Moulodi, affirms that this manuscript is an honest, accurate, and transparent account of the study being reported; that no important aspects of the study have been omitted; and that any discrepancies from the study as planned (and, if relevant, registered) have been explained.

## Supporting information

Supporting File

## Data Availability

The authors confirm that the data supporting the findings of this study are available within the article and in Supporting Information S1.
