# Supplementary figures and images for: Complications After the Use of Trans‐Tibial Prostheses in Patients With Diabetes: A Cross‐Sectional Study
Source: Health Sci Rep. 2026 May 18;9(5):e72535. doi: 10.1002/hsr2.72535 (PMC13183775; doi:10.1002/hsr2.72535)

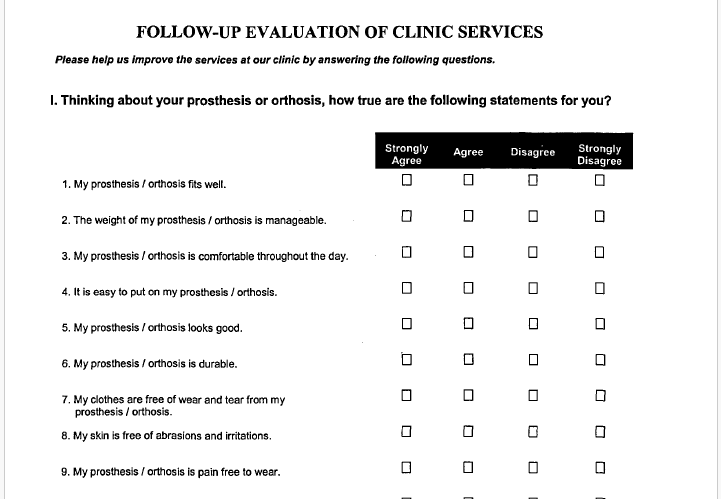

Supplement: Supplementary file 1 — Supporting File [file HSR2-9-e72535-s001.docx]
